# The Scarless Neo-Umbilicus in DIEP-Flap Breast Reconstruction

**DOI:** 10.3390/jpm13020315

**Published:** 2023-02-11

**Authors:** Sora Linder, Hisham Fansa

**Affiliations:** 1 Department of Plastic Surgery and Breast Center Zürich, Spital Zollikerberg, Zollikerberg, 8125 Zürich, Switzerland; 2 Department of Plastic, Reconstructive, and Aesthetic Surgery, Hand Surgery, Klinikum Bielefeld, OWL-University, 33604 Bielefeld, Germany

**Keywords:** DIEP-flap, breast reconstruction, abdominoplasty, umbilicus, umbilicoplasty, neo-umbilicus, donor site aesthetics

## Abstract

While the DIEP (deep inferior epigastric perforator) flap has become the gold standard in autologous breast reconstruction due to its favourable tissue characteristics and preserved abdominal wall function, a constant attempt is done to improve the outcome of the donor site. Even if just a small detail, the umbilicus has a big impact on the overall aesthetic outcome of the donor site. As an already established technique in abdominoplasties, we introduced the neo-umbilicus as the standard procedure for DIEP donor site closure. The aim of this study was to assess the aesthetic outcome of this neo-umbilicoplasty technique in DIEP-flaps. This is a single-center cohort study. A total of 30 consecutive breast cancer patients were treated during a period of 9 months with a mastectomy and an immediate reconstruction with a DIEP-flap. In all patients, the reconstruction of the umbilicus was done by an immediate neo-umbilicoplasty technique, consisting of a cylindrical fat resection at the new loco typico and fixation of the dermis directly to the rectus fascia. All patients were photographed in a standardised setting. Subjective patient satisfaction was assessed with a survey consisting of three questions and aesthetic outcome was evaluated by an independent professional panel consisting of three plastic surgeons. The results were compared to a previous cohort of conventional umbilicoplasties in DIEP-flap patients. Twenty-six patients participated in the follow-up study. There were no wound complications associated with the neo-umbilicus. Questionnaire results demonstrated high but not statistically significant different patient satisfaction. The panel scores were statistically significant (*p* < 0.05) better for the neo-umbilicus reconstructions. The aesthetic outcome was rated higher in patients with a higher BMI compared to those in patients with a low BMI. The creation of a neo-umbilicus at the donor site after DIEP-flap breast reconstruction is a quick and safe technique and leads to a superior aesthetic donor site result.

## 1. Introduction

The umbilicus is a very small but important aesthetic landmark of the abdominal wall and it greatly contributes to the aesthetic appearance. It is usually a round depressed structure with a diameter from 1.5 to 2 cm [1]. The natural location is at the crossing of the midline (linea alba) and the line transecting the superior iliac crests [2]. 

The absence of an umbilicus is very obvious and leads to an unnatural abdominal appearance, a thick scar around the umbilicus or a misplaced umbilicus contributes to an “operated look” [3].

The deep inferior epigastric perforator flap (DIEP) has become the widely accepted gold standard for breast reconstruction. Its benefits are a low donor site morbidity and due to the tissue characteristics, a superior aesthetic result of the reconstructed breast [4]. Unlike in the pioneering days of microsurgery, autologous breast reconstruction today is not only about the survival of the flap but also about the aesthetic result of the breast and the donor site. The removal of tissue from the abdomen makes it necessary to re-inset or reconstruct the umbilicus. Although in recent studies additional attention is drawn to the donor site aesthetics [5], the little detail of the reconstructed umbilicus has not been discussed in detail. The technique of a neo-umbilicus has been described by several authors in the context of abdominoplasty surgery. Especially after the loss of the umbilicus due to infection, repair of umbilical hernia, urachal cyst repair, omphalocele repair, gastroschisis repair, and tumor excision neo-umbilicoplasty is used [6,7]. The techniques vary but mainly consist of different cutaneous flaps that are attached to the rectus sheath by an external incision. Hoyos et al. for example use a delayed technique with defatted X-shaped flaps to create a new umbilicus after extended lipo-abdominoplasty. Like the conventional technique, this produces scars [8].

In conventional umbilicoplasty, the umbilicus is left in its original position. While harvesting the DIEP-flap the umbilicus is cut in a circular fashion. An inferiorly open “U” is excised at the inferior base. Correspondingly, an inferior pedicled “U” flap is cut from the abdominal wall at the umbilical position. A 3-0 Vicryl suture is used to attach this flap to the inferior base of the umbilicus where the “U” was removed, and both structures are anchored to the rectus fascia. Thus, the umbilicus is fixed to the abdominal wall. 

**Figure 1:**
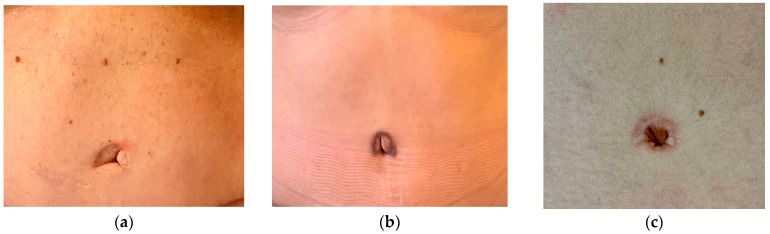
Conventional umbilicoplasty with an inferior flap from the abdominal wall in DIEP-flap reconstructions. The flap is secured to the base of the umbilicus at the rectus sheath. (**a**) Inconspicuous scar but protrusion of the umbilicus; (**b**) periumbilical scar visible due to pigmentation. (**c**) Hypertrophic scar after secondary healing with a darker color in the umbilicus.

We have noticed some unfavourable results after regular umbilical inset in abdominoplasty and after DIEP-flap harvest (Figure 1). This led us to adopt a new technique of umbilicoplasty by removing the umbilicus and creating a new umbilicus from the abdominal wall. In the beginning, we followed the technique of Youssif which creates a central incision and uses four transcutaneous stitches [9]. We then abandoned the transcutaneous stitches/incision and switched to a buried fixation purely from the inside as described below. The aim of our study was to assess the result of a neo-umbilicus in comparison to the conventional technique of umbilicoplasty in creating a deeper and more natural-looking umbilicus without external scars.

## 2. Materials and Methods

This is a single-center cohort study evaluating the outcome of the newly implemented neo-umbilicoplasty in DIEP-flaps. The study was approved by the review board committee of our institution in Switzerland in 2021. We applied the technique of creating a neo-umbilicus since the end of 2021 and operated on 30 patients accordingly. A total of 30 consecutive breast cancer patients were treated during a period of 10 months with a mastectomy and an immediate reconstruction with a DIEP-flap. All patients underwent immediate reconstruction of the umbilicus by a neo-umbilicoplasty technique that was described by Yousif et al. [9]. In the first 4 patients, we performed transcutaneous stitches and made a short incision in the center of the neo-umbilicus to ensure very stable mattress sutures. The sutures were performed with 5-0 Prolene, but unfortunately, remained visible for a very long time. Therefore, we modified the technique to fit our purposes, see below. To evaluate the results of the new technique, the outcome of the 30 patients was compared to the previous 30 patients who received a DIEP-flap with the conventional technique of insetting the original umbilicus with a caudally pedicled U-flap.

**Figure 2:**
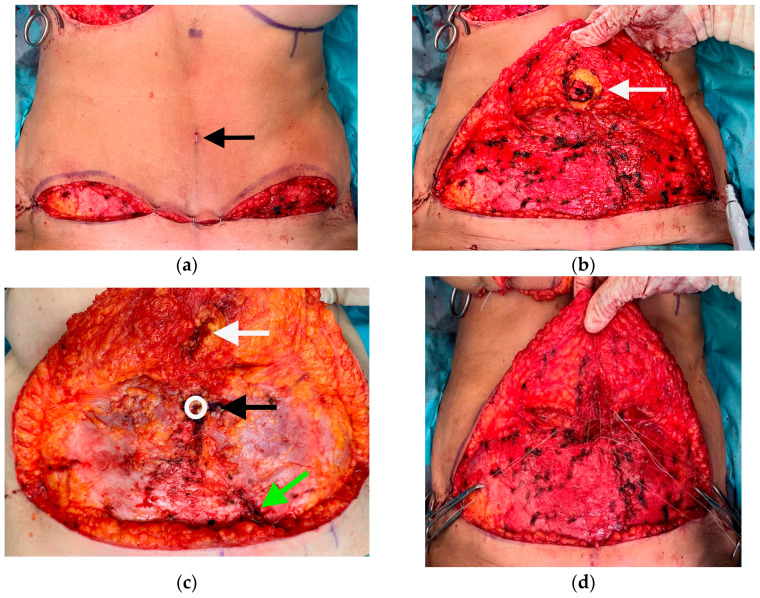
(**a**) Intraoperative marking of the new position of the umbilicus on the abdominal wall with a stapler (arrow) after temporary closure. (**b**) Cylindric resection (arrow) of the abdominal wall tissue up to the dermis. (**c**) Intraoperative situs; black arrow: position of the new umbilicus on the rectus sheath after resecting the umbilicus, sutures will be placed clockwise in the 3,6,9, and 12 positions. White arrow: corresponding resected fat cylinder from the abdominal wall. Green arrow: closure of rectus sheath after DIEP-pedicle harvest. (**d**) Clockwise prepared 3-0 Vicryl sutures in the 3,6,9, and 12 positions.

**Figure 3:**
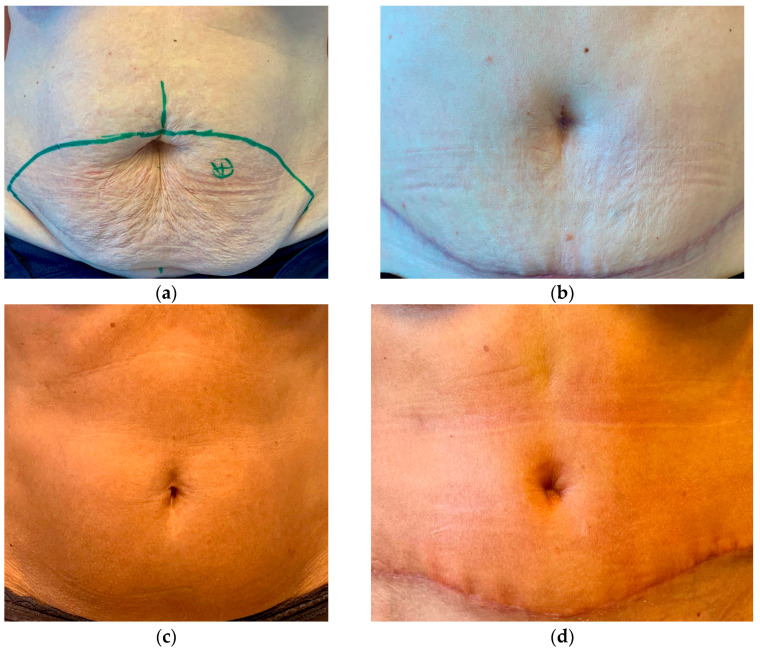
(**a**,**b**) Patient pre-(**a**) and postoperative (**b**) after neo-umbilicoplasty. (**c**,**d**) Another patient pre-(**c**) and postoperative (**d**).

Neo-umbilicoplasty: After elevation of the flap, resection of the umbilicus, closure of evident hernia, and dissection of the upper abdominal wall in a limited dissection technique up to the xyphoid process according to Lockwood [10], the patient is placed in a beach chair position with a bent hip and a temporary closure of the wound is performed. The position of the neo-umbilicus is defined according to the anatomic landmarks: the new position is at the linea alba at the level of the superior iliac crest. In most cases, it is close to the old umbilicus position. At the same time, the donor site scar in the lower abdomen is taken into consideration and enough distance from the umbilicus to the abdominal scar is preserved. However, we did not define a standard distance between the abdominal scar and the new umbilicus position. The new position of the umbilicus is marked with a stapler clip on the abdominal wall. The temporary closure is reversed and on the corresponding inside of the abdominal wall, a cylindrical fat resection is performed up to the dermis. Buried 3-0 Vicryl mattress sutures at 12-, 3-, 6-, and 9 o’clock positions fixate the dermis without penetrating the skin to the surface directly to the rectus fascia at the new loco typico. The 12 o’clock stitch is placed in an upward position to develop some kind of hooding (Figure 2). In addition, the abdomen is closed with quilting sutures in the linea alba, and lateral to the rectus abdominis muscle after the application of ropivacaine. No drainage is necessary (Figure 3).

A fat gauze is rolled up and placed in the new concavity, only fixated with an overlying band-aid. As the rectus sheath was opened for the elevation of the DIEP-flap, the patients need to wear compression pants that apply constant pressure on the umbilical bandage and secure its position. The compression pants have to be worn for a total of 6 weeks and sports activities have to be avoided for the same period. 

The patients were photographed in a standardized setting. Subjective patient satisfaction was assessed 5 months after surgery with a digital survey consisting of 3 questions: Are you satisfied with the appearance of your umbilicus? Does the umbilicus look natural? Are you satisfied with the colour of the skin in the umbilicus? The answer was either yes or no. The aesthetic outcome was additionally evaluated by an independent professional panel consisting of 3 external plastic surgeons who are experienced with DIEP-flap surgery but were not involved in the treatment of our patients. The surgeons received pictures of 12 conventionally operated umbilici and 12 neo-umbilici. The rating was done on a scale from 1 to 5, with 1 being a poor result and 5 being a very good result.

Statistics were performed with GraphPad Prism Version 8.4.3. (Boston, MA, USA). To compare the results of the patients’ questionnaire a two-sided exact Fisher test was used. To analyze the surgeons’ evaluation a two-sided Mann–Whitney-U test was used. An error probability of *p* < 0.05 was considered statistically significant.

## 3. Results

We had no wound-healing problems associated with the neo-umbilicus and no infections. In a few cases where the patients were very slim, the concavity remained shallow and the rim of the neo-umbilicus remained poorly defined. The base of the neo-umbilicus was of the same colour as the surrounding abdominal wall skin, which added to the inconspicuous appearance of the new umbilicus. 

Twenty-six patients participated in the follow-up study (Table 1). Twelve patients from the neo-umbilicoplasty group and fourteen patients from the conventional group responded to the invitation. The questionnaire results demonstrated good patient satisfaction in both groups. The neo-umbilicoplasty showed slightly better results, but the results did not reach significance (1.–3. question: *p* = 0.68; *p* = 0.70, *p* = 0.65). 

The aesthetic outcome was rated higher in patients with a higher BMI compared to those in patients with a lower BMI. The slim patients were satisfied with the result of the neo-umbilicus; however, a better definition of the umbilical features would have been appreciated but was negligible due to the fact that the reconstruction of the breast was the focus of the patients. With the consecutive application of this new technique in DIEP patients a learning curve could be observed. In the beginning, the vertical skin incision described in the work of Yousif et al. [9] was executed, but later omitted as access from the inside was always given in our patient collective. The bolster bandage that was applied in the original work and that was fixated via a tie-over suture was omitted as well, as the compression pants can equally fulfill the purpose, and the risk of infection is then reduced. No further operations were needed for umbilical corrections.

**Figure 4:**
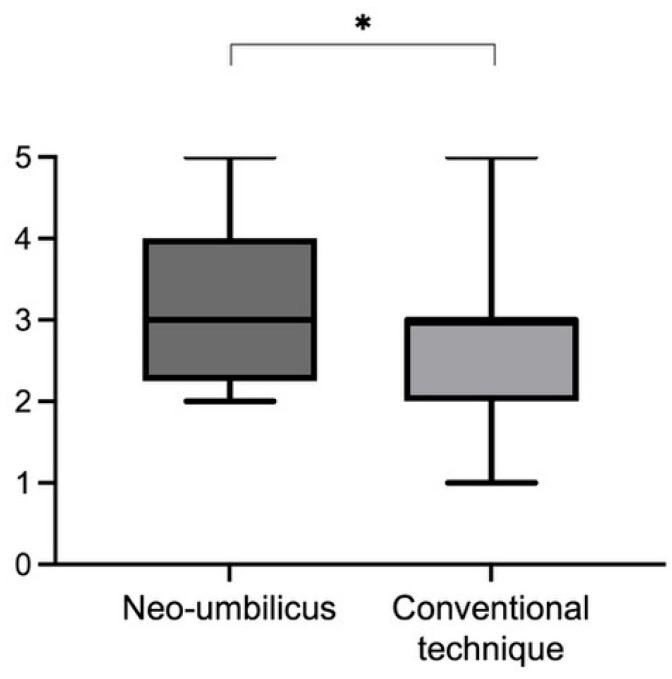
Rating of a panel of plastic surgeons. The rating was done on a scale from 1 to 5, with 1 being a poor result and 5 being a very good result. The neo-umbilicus scored significantly better. The mean result for the new technique was 3.3 (5-2, SD 1.069) compared to 2.667 (5-1, SD 1.095) for the conventional technique. This difference between techniques was significant (* *p* = 0.016).

**Figure 5:**
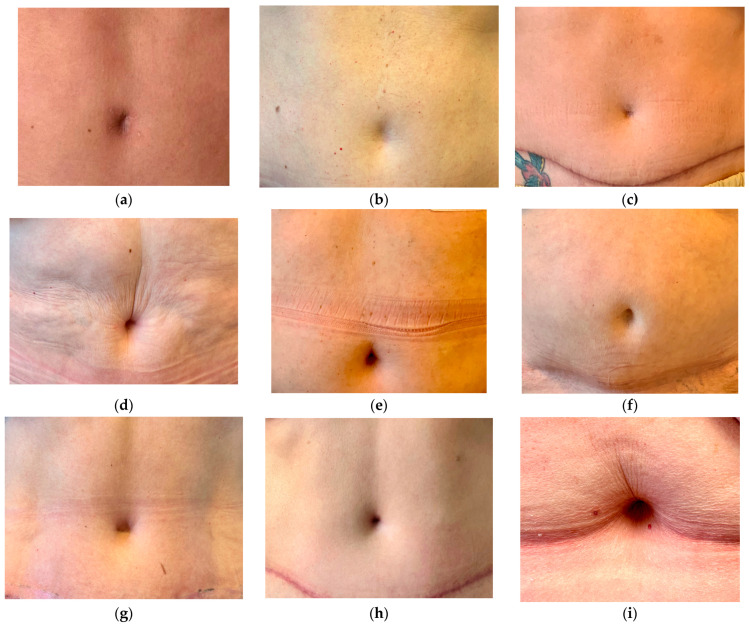
Results after neo-umbilicoplasty. (**a**) In the beginning, the technique of Yousif [9] was used. The transcutaneous stitches can cause visible scarring. (**b**,**c**) Less defined umbilicus in skinny patients and due to wide resections at the beginning of the technique. (**d**–**i**) Patients with good results after neo-umbilicoplasty. Modification to scarless technique with sutures between the rectus sheath and the abdominal wall dermis. The technique produces a scarless umbilicus. Hooding develops over time.

All plastic surgeons from the panel rated the neo-umbilicoplasty better compared to the conventional technique (Figure 4). The mean result for the new technique was 3.3 (5-2, SD 1.069) compared to 2.667 (5-1, SD 1.095) for the conventional technique. The result was significant (*p* < 0.05), with higher ratings for the neo-umbilicoplasty. The surgical results are shown in Figure 5.

## 4. Discussion

With the DIEP-flap being the gold standard in breast reconstruction due to its very good tissue characteristics match and its reliability, the abdominal wall has been used as the main harvest area in this patient collective. Patients often find it easier to accept the “additional wound area” as they hope for an improvement of the abdominal wall appearance [11]. Unfortunately, little attention has been given to the restoration of the abdominal wall after the harvest of the flap, so some patients are left disappointed with the donor site result. Although seen as one of the basics of plastic surgery, cosmetic abdominoplasty has been associated with a lot of disappointed patients and litigation for surgeons [12]. Several studies highlighted the importance of donor site aesthetics [5,13], but only a few papers suggest any change of technique for the small detail of the reconstructed umbilicus. In the context of cosmetic abdominoplasties and reconstructions of the abdominal wall due to hernia, etc. A vast variety of umbilical reconstructions have been discussed. The sequelae of classical umbilicoplasty often include bulky, hypertrophic, and/or constricted scars, very sharp edges, and an unnatural operated appearance. Moreover, in many cases, the color of the umbilicus does not match the skin color of the abdomen. Therefore, especially in the context of aesthetic procedures efforts are made to improve the result and make the umbilicus less conspicuous and the technique of the neo-umbilicus was reintroduced [8].

In abdominoplasty or DIEP-flaps, the poorly healed umbilicus can be an aesthetic stigma. There were also results in our patients’ cohort that were described as not good. For example, only 57% of patients with the conventional technique report that the umbilicus looks natural. Although the reconstruction of the breast is the focus, the aim should be that the donor site looks as aesthetically pleasing as possible in order to increase the acceptance of the reconstructive surgery and not to cause secondary problems for the patients. With the neo-umbilicoplasty, we were able to improve the patient’s assessment. Although this patient-reported outcome was not significantly different, this may be methodological. A point that was not assessed is sensitivity. Most patients after conventional umbilicoplasty complain about a loss of sensitivity. This may attribute to an unfavorable assessment as the part is not “felt”. It is also possible that our assessment 5 months after the procedure is too early, as some people do not recover sensitivity so early, and hooding of the umbilicus is also seen later improving the aesthetic result. In any case, the assessments of the independent panel of plastic surgeons familiar with DIEP-flap surgery show a significant improvement with neo-umbilicoplasty. 

While we were establishing this technique as our operative standard in DIEP-flap reconstruction, we were confronted with “self-inflicted” problems. The initial hole in the abdominal wall after cylindrical fat resection was too small and the patient was complaining postoperatively about a point-like unnatural umbilicus. The stitches were not positioned exactly and the edges were not clearly defined so that especially in skinny patients the umbilicus looked like it got lost. Therefore, we applied an upward placement of the 12 o’clock stitch to develop some kind of hooding. The thicker the abdominal wall is, the wider the resected cylinder should be, providing a suitable umbilicus. 

In the beginning, we performed transcutaneous stitches and made a short incision in the center of the neo-umbilicus to ensure very stable mattress sutures. The sutures were performed with 5-0 Prolene, but unfortunately, remained visible for a very long time. Therefore, we modified our technique to tangential dermal stitches from the inside without skin penetration. With these adjustments, the neo-umbilicoplasty results became very reproducible, persistent, and pleasing. Additionally, there are no visible scars. 

The advantages of this technique are (1) fewer scars and consecutively lower risk of scar contracture, hypertrophic scarring, and contraction into a circle, which are major setbacks in the conventional umbilicoplasty results. (2) The technique is applied with ease, it is reliable, and leads to consistent, reproducible results after an initial steep learning curve. The procedure is quick and does not lead to a prolongation of the donor site closure compared to the application of conventional umbilicoplasty. (3) Fewer wound healing problems, especially fewer infections are observed than with the conventional technique. The neo-umbilicus complements our technique of quilting sutures in the linea alba. This reduces seromas and allows drainage to be omitted. A delay in adjuvant chemotherapy is avoided. (4) Postoperative wound care involves little effort for patients who are already occupied with the new breast and abdominal wall. (5) The position of the new umbilicus can be adjusted in consideration of the donor site scar and can be placed slightly higher up. (6) Basically, all patients appreciate the improvement of the abdominal wall appearance and having an umbilicus after the reconstructive operation.

Additionally, in obese patients, patients prone to wound healing complications (e.g., diabetes, obesity), or patients with abdominal scars the neo-umbilicoplasty can be postponed for secondary reconstruction. The DIEP-flap is harvested and the adjacent tissue including the original umbilicus is removed. Healing is safer as cranial undermining is not necessary and direct abdominal wound closure can be performed. Then the umbilicus can be secondarily reconstructed as originally described by Yousif et al. or other techniques with an external approach [8,14]. 

Disadvantages are (1) the risk of unnaturally pigmented scars when the skin is penetrated and (2) the risk of the poor definition of the umbilical edges in very skinny patients if the cylinder is made too big. In these patients, the upward placement is important to develop some kind of hooding, but the learning curve for this technique is slower. Additionally, the resected fat cylinder in these patients should be no wider than 1.5 cm.

## 5. Conclusions

The creation of a neo-umbilicus at the donor site after DIEP-flap breast reconstruction is a quick and safe technique and leads to a superior aesthetic donor site result compared to our conventional technique.

## Figures and Tables

**Table 1:** Results of the patients’ questionnaire. The neo-umbilicus received better ratings, but this was not significant.

Questions	Neo-Umbilicus (12 Patients)		Conventional Technique (14 Patients)	
Are you satisfied with the umbilicus?	75% yes	25% no	64 % yes	36% no
Does it look natural?	67% yes	33% no	57% yes	43% no
Are you satisfied with the skin colour?	83% yes	17% no	71% yes	29% no

## Data Availability

Supporting data is available from the authors upon request.
